# Forme pseudo tumorale d'une pneumopathie chronique à éosinophiles d’évolution fatale

**DOI:** 10.11604/pamj.2015.20.154.6270

**Published:** 2015-02-18

**Authors:** Nabil Hammoune, Faycal El Guendouz, Siham Elhaddad, Hicham Janah, Abdelaziz Hommadi

**Affiliations:** 1 Service de Radiologie, Troisième Hôpital Militaire, Laayoune, Maroc; 2 Service d'Endocrinologie, Troisième Hôpital Militaire, Laayoune, Maroc; 3 Service de Radiologie, Centre Hospitalier Universitaire Ibn Sina, Rabat Maroc; 4 Service de Pneumologie, Troisième Hôpital Militaire, Laayoune Maroc

**Keywords:** Pneumopathie chronique idiopathique à éosinophilies, hyperéosinophilie, maladie de Carrington, Idiopathic chronic eosinophilic pneumonia, hypereosinophilia, Carrington disease

## Abstract

La pneumopathie chronique idiopathique à éosinophile est une pathologie rare, de cause inconnue, caractérisée par des opacités pulmonaires périphériques, une éosinophilie périphérique >1000/mm^3^ et /ou une éosinophilie alvéolaire >25%. Le diagnostic est difficile à cause de la non spécificité des signes cliniques et radiologiques. Le traitement se base essentiellement sur la corticothérapie. L’évolution est généralement favorable. Nous rapportons un cas de cette entité rare dans sa forme pseudotumorale sans hyperéosinophilie, de diagnostic tardif suite à l’étude histologique de la lobectomie chirurgicale et d’évolution fatale.

## Introduction

la pneumonie chronique idiopathique à éosinophiles ou maladie de Carrington est une maladie rare de cause inconnue, caractérisée par l'association de symptômes respiratoires et généraux subaigus ou chroniques, une éosinophilie alvéolaire et/ou sanguine et des infiltrats pulmonaires de topographie périphérique en imagerie. Il s'agit d'une pathologie bénigne d’évolution favorable. Nous rapportons un cas de cette pathologie rare dans sa forme pseudotumorale de diagnostic difficile caractérisé par l'absence d’éosinophilie sanguine et alvéolaire et d’évolution fatale. Nous rappellerons aussi les caractères cliniques, radiologiques, thérapeutiques et évolutifs de la maladie de Carrington à travers une revue de littérature.

## Patient et observation

Patient âgé de 64 ans, diabétique de type 2 sous antidiabétiques oraux, ayant comme antécédent une tuberculose pulmonaire traitée sans séquelles, une rhinite allergique saisonnière et un tabagisme chronique, qui a rapporté un mois avant son admission une toux productive et des hémoptysies évoluant dans un contexte de fièvre et altération de l’état général. L'auscultation pleuro-pulmonaire a révélé des râles crépitants au champ pulmonaire droit, l'examen cardiovasculaire est sans anomalie, le reste de l'examen physique est sans particularités. Le bilan biologique initial a mis en évidence une anémie hypochrome microcytaire, un taux de leucocyte normal sans hyper éosinophilie sanguine et une CRP à 171mg/l.

**Figure 1:**
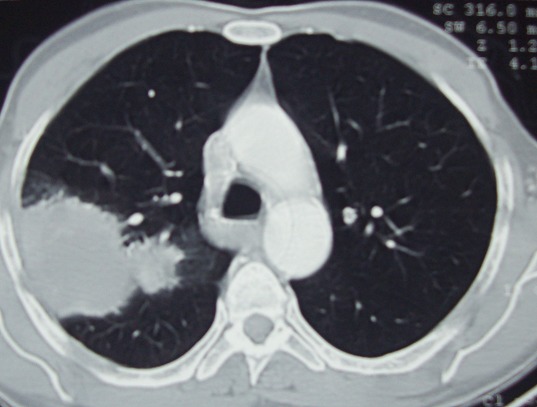
TDM thoracique en coupes axiales et en fenêtre parenchymateuse montrant condensation apicale droite, homogène, présentant une large base d'implantation pleurale

La radiographie thoracique de face a montré une opacité homogène apicale droite, le scanner sans et avec injection du produit de contraste a objectivé une condensation apicale droite, homogène, présentant une large base d'implantation pleurale sans épanchement pleural ni d'adénopathie médiastinale ou de lésion parenchymateuse pulmonaire homo ou controlatérale (Figure 1). La fibroscopie bronchique a trouvé un bourrelet du segment dorsal de la lobaire supérieure droite et l’étude cytologique du liquide d'aspiration bronchique a conclue à une hyperplasie des cellules ciliées sans cellules suspecte de malignité et sans hyperéosinophilie. L’étude anatomopathologique n'a pas trouvé de signe de malignité. La recherche de bacille de Kokh dans les crachats et le liquide d'aspiration bronchique était négative.

**Figure 2:**
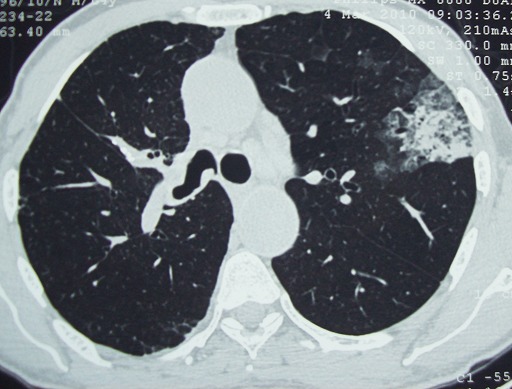
TDM thoracique en coupes axiales et en fenêtre parenchymateuse montrant un infiltrat apical gauche

Le patient a été mis sous traitement antibiotique à base d'amoxicilline, d'acide clavulanique et de ciprofloxacine mais sans amélioration clinique ni radiologique. Le malade a représenté par la suite deux épisodes d'hémoptysie de grande abondance ayant nécessite une prise en charge chirurgicale. Il a bénéficié d'une lobectomie apicale droite. L’étude anatomopathologie a trouvé un tissu inflammatoire essentiellement mononuclée avec présence d'amas de polynucléaires éosinophiles dans la lumière des alvéoles. Devant ce tableau clinico-radiologique et anatomopathologique le diagnostic d'une pneumopathie à éosinophile a été évoqué. Une anamnèse dirigée a été reprise à la recherche d'une cause de cette pneumopathie à éosinophile, il n y avais pas de prise médicamenteuse ni de séjour en endémie parasitaire, les parasitologies de selle étaient négatives, les tests cutanés ainsi la sérologie aspergillaire étaient négatifs, le bilan immunologique en particuliers les ANCA était négatif, Les échographies abdominale et cardiaque pratiquées à la recherche d'atteinte extrapulmonaire étaient normales. Le diagnostic de pneumopathie chronique idiopathique à éosinophilies a été retenu. Le malade a été mis sous traitement corticoïde avec une évolution remarquablement favorable. Après 15 mois de surveillance, il a présenté une récidive de sa symptomatologie clinique initial avec apparition d'un infiltrat pulmonaire périphérique apical du coté controlatéral au scanner thoracique (Figure 2), l’évolution a été marqué par une altération rapide de l’état général, le malade est décédé en réanimation suite à un état de choc hémodynamique par hémoptysie foudroyante.

## Discussion

La pneumopathie chronique idiopathique à éosinophiles (PCIE), aussi appelée maladie de Carrington, du nom de l'auteur qui en fit la première description en 1969, s'inscrit dans le cadre des pneumopathies à éosinophile sans cause déterminée [1]. Elle correspond à des infiltrats pulmonaires à prédominance périphérique, associées à une éosinophilie circulante et/ou alvéolaire [2]. D’évolution chronique, cette maladie rare, représente moins de 2,5% de l'ensemble des pneumopathies infiltrantes diffuses, elle touche préférentiellement la femme d’âge moyen. Dans toutes les séries publiées, on retrouve souvent un contexte atopique avec antécédent d'asthme, chez deux tiers des patients, qui peut précéder la maladie de plus de 20 ans, c’était le cas pour notre patient qui était suivi pour rhinite allergique [3]. Le diagnostic positif est retenu devant l'association d'un faisceaux d'arguments: la présence de symptômes respiratoires et le plus souvent généraux à caractère subaigu ou chronique, la présence d'une éosinophilie sanguine et/ou alvéolaire, la présence d'opacités le plus souvent de nature alvéolaire en imagerie thoracique et l'exclusion de toute cause déterminée de pneumopathie éosinophilique parasitaire, en particulier une helminthiase ou une étiologie médicamenteuse [4]. Mais cette définition est trop restrictive: les images radiologiques sont plus diversifiées; l’éosinophilie sanguine peut faire défaut ou être transitoire, de plus elle s'efface vite sous corticothérapie, masquant le diagnostic pour un temps variable [5]. La symptomatologie clinique décrite dans la littérature est comparable à celle qu'a présenté notre patient, elle est dite non spécifique, elle associe des signes respiratoires à type de toux, dyspnée intense, douleurs thoraciques et des signes généraux à type de fièvre, d'amaigrissement et de sueurs sans atteinte extra respiratoire [4, 6].

Le cliches du thorax montre habituellement des opacités alvéolaires, de pourtours flous, non systématisées, périphériques et labiles, réalisant l'aspect classique négatif d’œdème pulmonaire mais ne sont pas spécifiques. Ces images intéressent surtout les lobes supérieurs et moyens [3, 6, 7]. La TDM thoracique a pour intérêt principal de mieux préciser la topographie périphérique des lésions lorsqu'elle n'est pas évidente sur la radiographie standard et la nature alvéolaire des opacités allant de l'aspect en verre dépoli à la condensation avec bronchogramme aérien. Les bronchectasies sont typiquement absentes. Des épaississements septaux, des opacités en bande sous pleurales et des adénopathies médiastinales sont parfois constatées, le scanner peut révéler des épanchements pleuraux le plus souvent peu importants qui passent donc le plus souvent inaperçus en radiographie standard [3, 4, 7]. C'est la formule leucocytaire qui conduit le plus souvent au diagnostic, l’éosinophilie périphérique sanguine est constamment très marquée. Elle est en règle générale supérieure à 1000 éléments/mm3, un syndrome inflammatoire biologique est fréquemment rapporté. Une augmentation du taux d'immunoglobulines E est présente dans la moitié des cas. L’éosinophilie alvéolaire est particulièrement élevée, supérieure à 40% [2]. Chez notre patient, l'absence d'hyperéosinophilie sanguine et alvéolaire était troublante, pouvait au contraire discuter une origine tumorale ou tuberculeuse, l'incertitude diagnostique et l'hémoptysie ont conduit à poser l'indication chirurgicale, dont l'analyse histologique a montré une infiltration d’éosinophilie dans l'interstituim et les lumières alvéolaires. Un cas pareil a été décrit par Marq et al dont le diagnostic était exclusivement histologique [8].

L'exploration fonctionnelle respiratoire n'est pas nécessaire au diagnostic, lorsqu'elle est réalisée, elle montre un trouble ventilatoire obstructif ou restrictif [2, 5]. En fait, le diagnostic de PCIE est un diagnostic d’élimination qui ne doit être retenu qu'après avoir éliminé toute pneumopathie hyperéosinophilique d'origine déterminée [7]. Il faut chercher systématiquement une étiologie médicamenteuse ou une origine parasitaire. Chez un sujet atopique, il convient de rechercher une aspergillose broncho-pulmonaire allergique. Devant des signes systémiques, il faut penser aux vascularites pulmonaires notamment le syndrome de Churg et Strauss et les vascularites à ANCA [4].

La PICE répond de façon spectaculaire à la corticothérapie orale, qui a même été proposée comme test diagnostique. L'amélioration clinique survient dans les 48h chez la plupart des patients, et la régression des images radiographiques en une semaine chez environ 70% des patients [3]. L’évolution est souvent favorable cependant, les récidives ou l’évolution vers un asthme sévères sont fréquente lors de la diminution ou l'arrêt du traitement, Des cas exceptionnels d’évolution vers une fibrose pulmonaire ont été décrit [7]. Le pronostic vital est favorable bien qu'un décès lié à une infection nosocomiale chez un patient ventilé présentant une pneumopathie à éosinophilies ait été décrite, l’évolution chez notre patient était malheureusement fatale par un état de choc hémodynamique suite à une hémoptysie foudroyante.

## Conclusion

La pneumopathie chronique idiopathique à éosinophiles est une pathologie rare, de signes clinico-radiologiques non spécifiques ce qui rend le diagnostic difficile. La confirmation du diagnostic peut avoir recours à une preuve histologique comme le cas de notre observation. C'est une maladie le plus souvent bénigne qui évolue favorablement sous corticoïde mais elle peut menacer le pronostic vital.
